# Transient infantile hypertriglyceridemia with jaundice: A case report

**DOI:** 10.1097/MD.0000000000025697

**Published:** 2021-04-30

**Authors:** Jun Wang, Fang Sun, Pengfei Xu, Yufeng Zhang, Xinrong Sun, Huiling Deng

**Affiliations:** a Second Department of Infectious Diseases; b Department of Respiratory, Xi’an Children's Hospital; c Xi’an Central Hospital, Xi’an, Shaanxi Province, China.

**Keywords:** glycerol-3-phosphate dehydrogenase 1, hepatic steatosis, transient infantile hypertriglyceridemia, hypertriglyceridemia, jaundice

## Abstract

**Rationale:**

Transient infantile hypertriglyceridemia (HTGTI) is a rare autosomal recessive inherited disease caused by inactivating mutations in the glycerol-3-phosphate dehydrogenase 1 gene. To date, only a few patients have been reported worldwide. The symptoms of the affected individuals present a certain degree of transient hypertriglyceridemia, hepatomegaly, elevated liver enzymes, persistent fatty liver and hepatic fibrosis in early infancy. However, the clinical characteristics and pathogenesis of this disease are remain unclear.

**Patient concerns:**

A one month and twenty-five days old girl was admitted to hospital because of persisted jaundice and hepatomegaly for fifty days.

**Diagnose:**

The girl was diagnosed with HTGTI coincident with a noval mutation in glycerol-3-phosphate dehydrogenase 1.

**Intervention:**

She was advised to take low-fat diet and supplement of medium-chain fatty acids.

**Outcomes:**

Her jaundice was gradually normal at the age of 4 months without any treatment, and hypertriglyceridemia were normal at the age of 13 months, but still had elevated transaminases and hepatic steatosis.

**Lessons:**

Jaundice may be a novel phenotype in HTGTI. The report contributes to the expansion of HTGTI's gene mutation spectrum and its clinical manifestations.

## 1 Introduction

Transient infantile hypertriglyceridemia (HTGTI) is a rare autosomal recessive inherited disease with lipid metabolic disorder and is caused by the inactivation and mutation of glycerol-3-phosphate dehydrogenase gene (GPD1) mapped to chromosome 12q12-q13. GPD1 gene encodes cytoplasmic NAD-dependent GPD1 and catalyzes the reversible redox reaction of dihydroxyacetone phosphate (DHAP) and nicotine adenine dinucleotide (NADH) to glycerol-3-phosphate (G3P) and NAD^+^, playing a critical role in carbohydrate and lipid metabolism.^[1]^ A homozygous mutation in GPD1 gene was firstly reported by Basel-Vanagaite et al. as the cause of HTGTI in 10 individuals from four consanguineous Israeli Arab families in 2012, and up to now there have been genetically confirmed 18 patients from 6 articles worldwide.^[2–7]^ The major clinical manifestations of the patients include moderate to severe hypertriglyceridemia, hepatomegaly, elevated transaminases, hepatic steatosis and fibrosis. However, the clinical characteristics and pathogenesis of this disease are remain unclear.

In this article, we reported a Chinese girl who was diagnosed with HTGTI by next-generation sequencing. She not only had major features of GPD1 deficiency, but also exhibited a new clinical symptom characterized by moderate jaundice and a novel compound heterozygous mutation those had never been reported before. This report expands the clinical manifestations and gene mutation spectrum of HTGTI. The research was approved by the Medical Ethics Committee of Xi’an Children's Hospital. Written informed consent for publication of clinical details were obtained from the patient's parents.

## 2 Case report

In April 2019, a Chinese girl of one month and twenty-five days old was admitted to our hospital because of persisted jaundice and hepatomegaly for fifty days. With a birth weight of 3.4 kg, she was born at gestational age of 40 weeks by spontaneous labor. The mother had a history of cold during pregnancy, and the family has no history of inherited metabolic disease. The patient is the second child of a non-consanguineous *Han* Chinese couple and has a healthy older sister. After birth, she was given mixed feeding and presented with normal growth and development.

The past medical history showed that she was diagnosed with neonatal hyper-unconjugated bilirubinemia at three days with a total bilirubin of 220 μmol/L. She was initially intermittently treated with probiotics and Yinzhihuang oral liquid (a Chinese herbal formula), antibiotics in local hospital for more than one month, however, her jaundice didn’t improve significantly. At one month and twenty-five days, she was transferred to our hospital.

The physical examination showed moderate yellowish skin and mucosa, and hepatomegaly (approximately 5.0 cm under the right costal margin). She had no abnormality in physical appearance and mental status, had no rash and purpura, her lungs, heart, nervous system and superficial lymph nodes were unremarkable. Laboratory investigation showed that serum total bilirubin (TB) was 233.8 μmol/L, indirect bilirubin (IB) was 205.3 μmol/L, and elevated fasting triglyceride was 6.36 mmol/L. Liver ultrasound revealed hepatic steatosis and hepatosplenomegaly. Further biochemical and pathogen investigstions were normal except slightly increased urine dicarboxylic acids. During this hospitalization, the patient was mainly diagnosed as indirect hyperbilirubinemia and mild bronchopneumonia and was given anti-infection and phenobarbital for 12 days. Under this regimen, her jaundice (TB 160.1 μmol/L, IB 129.4 μmol/L) and fasting triglyceride (1.4 mmol/L) had been improved a little, but the hepatosplenomegaly persisted, alanine aminotransferase was moderately higher and total bile acids slightly went up.

We speculated that she might have inherited metabolic disease, so the genetic testing was performed after obtaining written informed consent from her parents. The peripheral venous blood of the proband and her parents were collected for genetic testing using next generation of whole genomic DNA. The captured libraries were sequenced using Illumina HiSeq, analyzed by Clinic Sequence Analyzer from WuXiNextCODE, and tested with SureSelect Human All Exon V5 kit (Agilent Technologies, Inc.), and the sequencing data was mainly analyzed by Sentieon. Variants were noted by VEP (Variant Effect Predictor, Ensembl73). Three databases including ClinVar, OMM and HGMD were employed to filter known and possible pathogenic variants, and other tools were adopted to predict the function of missense mutation and to note non-coding sequence. These tools are large-scale sequencing databases including 1000 Genome Project, Exome Sequencing Project, Exome Aggregation Consortium and Genome Aggregation Database of Tokyo and the Netherlands. The result identified a heterozygous mutation NM-005276:exon7:c.901 G>T(p.E301X) in chr12:50501861 from the proband's mother. Family trio sequencing found the heterozygous mutation in the proband's mother, and Next Generation Sequencing predicted possibility of GPD1 heterozygosity deficiency of the proband and her father. This deficiency was an unreported compound heterozygous mutation in those databases. The patient's total bilirubin was gradually normal at the age of 4 months without any treatment. At the age of 7 months, the patient's gene sequencing showed a heterozygous mutation in GPD1 with a short fragment heterozygous deficiency from her father and a missense mutation C.901G>T (p.E301X) from her mother. The child's parents and older sister were asymptomatic. Taken into account of her clinical features, she was diagnosed with HTGTI, then was advised to take low-fat diet and supplement of medium-chain fatty acids. The patient's triglyceride were gradually normal at the age of 13 months. At the age of 16 months, she showed normal growth and no hepatosplenomegaly, but still had elevated transaminases and hepatic steatosis (See Table 1, which is monitoring result of liver function and blood during the follow-up year).

**Table 1:** Monitoring results of liver function and blood lipid during the follow-up years.

		Age						
Items	Normal value	1 mo 13 d	1 mo 26 d	2 mo 17 d	3 mo 14 d	4 mo 27 d	7 mo 10 d	1 yr 4 mo
TBIL	2.0–20.4 μmol/L	258.8	233.8	34.6	28.3	33.8	12.9	13.6
DBIL	0–6.8 μmol/L	10.9	28.5	9.9	9.0	13.3	5.2	4.1
IBIL	0–17.0 μmol/L	247.9	205.3	24.7	19.3	20.5	7.7	9.5
TP	55.0–7.0 g/L	62.2	73.5	71.0	60.2	70.6	67.4	72.0
ALB	36.0–50.0 g/L	43.1	49.9	53.0	45.3	44.1	48.1	48.8
ALT	4.0–35.0 U/L	106.0	34.0	24.0	73.0	187.0	91.0	95.0
AST	10.0–50.0 U/L	60.0	78.0	56.0	81.0	234.0	150.0	111.0
GGT	1.0–132.0 U/L	165.0	273.2	241.4	152.3	186.3	141.3	122.1
TBA	0–13.0 μmol/L	58.9	73.6	59.7	28.7	52.9	37.7	7.0
TCHO	2.8–5.2 mmol/L	NA	3.4	5.3	3.0	3.2	2.7	3.9
TG	0.56–1.7 mmol/L	NA	6.4	5.9	4.6	9.2	2.1	1.4
HDL-C	1.04–1.5 mmol/L	NA	0.6	0.8	0.5	0.6	1.1	1.4
LDL-C	2.1–3.4 mmol/L	NA	1.6	2.6	2.3	2.0	1.6	2.2
Glu	3.5–5.6 mmol/L	NA	1.9	2.9	3.9	4.5	4.1	4.0

## 3 Discussion

Hypertriglyceridemia can be caused by not only triglyceride synthesis, storage and degradation deficiency resulted by inherited inborn error of metabolism, but also secondary obesity, diabetes, liver diseases, hypothyroidism, etc.^[8]^ GPD1 gene encodes cytoplasmic NAD-dependent GPD1 and catalyzes the reversible redox reaction of DHAP and nicotine adenine dinucleotide (NADH) to G3P and NAD^+^, playing a crucial role in carbohydrate and lipid metabolism,^[1]^ its mutation is one of the major causes for primary hypertriglyceridemia with onset in infancy.

HTGTI has been described in a total of 18 individuals from 6 reports, whose onset ages range from birth to 7 years old. These patients harbored shared clinical features in the early period including significant hepatomegaly, elevated liver enzymes, fasting hypertriglyceridemia and hepatic change. A few of them presented with splenomegaly, elevated cholesterol, abnormal lipoprotein level, fasting ketotic hypoglycemia, intrahepatic cholestasis, dicarboxylic aciduria, liver cirrhosis, and slow growth and development. They have normal bilirubin and hepatic synthesis, as well as most of laboratory test results.

Our patient resembled the others with shared clinical characteristics of HTGTI, but also presented with moderate jaundice, although her jaundice gradually disappeared at 4 months after birth. After ruling out other causes, we consider jaundice as HTGTI's novel clinical manifestation, this might be related to disorder of bilirubin metabolism caused by steatosis of hepatocyte associated with hypertriglyceridemia. That is, a variety of endogenous substances in the body can affect the expression and activity of UDP-glucuronosyltransferase (UGT). Evidence showed that unsaturated fatty acids, bile acids, phospholipids and other substances are potent inhibitors of UGT,^[9]^ which may reduce the ability of UGT to bind indirect bilirubin. Another study verified that obesity- or hepatic steatosis induced UGT activity, steatosis was accompanied by increased hepatic triglyceride and free acid content and subsequently induced the network of transcriptional factors of aryl hydrocarbon receptor (AhR), constitutive androstane receptor (CAR), nuclear factor (erythroid-derived 2)-like 2 (Nrf2), pregnane Xreceptor (PXR), and peroxisome proliferator-activated receptor-α (PPAR-α) and then increased Ugt isoform mRNA expression. Because obesity-induced UGT expression in mouse liver and the induction was associated with AhR, CAR, PPAR-α, PXR, Nrf2, and peroxisome proliferator-activated receptor-γ coactivator-1α (PGC-1α) up-regulation,^[10]^ this may have a significant impact on the metabolism of bilirubin at different stages of hypertriglyceridemia. Our reported case had different GPD1 short fragment deficiency of compound heterozygous mutation C.901G>T(p.E301X) compared with other reported cases. The patient had a novel mutation site and type in comparison with previously reported 18 individuals harboring GPD1 homozygous mutation c.361-1G>C, c.806G>A (p.Arg269Gln), C.640T>C (p.Cys214Arg), c.523C>T, c.895G>A (p.G299R), or compound heterozygous mutation c.220-2A>g (Ala274Thr)+c.820G>A and R229Q+.

Base *et al.*^[2]^ suggested that the severe but transient hypertriglyceridemia caused by GPD1 mutation can increase the amount of hepatic G3P available for triglyceride synthesis. Alternatively, GPD1 is oriented toward the transformation from DHAP into G3P under physiological conditions, when this process was impaired, the accumulation of DHAP could induce over production of methylglyoxal, a celluarly highly toxic compound, which leads to renal fibrosis in diabetes patients. Hypertriglyceridemia could be related to increased hepatic synthesis of triglyceridemia. Furthermore, the transience of hypertriglyceridemia might follow the pattern of hepatocyte triglyceridemia, that is, a higher secretion rate in the new born over adults. Though there is a decline of hypertriglyceridemia during the end of infancy or childhood, hepatomegaly, elevated transaminases, fatty liver and liver fibrosis remain.^[7]^ The long-term impact of the disease is unclear as most affected patients are young individuals.

## 4 Conclusion

In summary, we report a Chinese HTGTI female patient with a noval mutation of GPD1 who presented with transient infantile hypertriglyceridemia, jaundice, hepatomegaly, elevated transaminases, and hepatic steatosis, providing references for the expansion of HTGTI's gene mutation spectrum.

## Author contributions

**Conceptualization:** Jun Wang, Huiling Deng.

**Data curation:** Pengfei Xu.

**Formal analysis:** Fang Sun.

**Investigation:** Yufeng Zhang.

**Writing – original draft:** Jun Wang, Pengfei Xu.

**Writing – review & editing:** Xinrong Sun, Huiling Deng.
